# Dialysis Disequilibrium Syndrome and Cerebellar Herniation with Successful Reversal Using Mannitol

**DOI:** 10.1155/2020/8850850

**Published:** 2020-08-25

**Authors:** Anna Curtis, Christian Lamb, Hussain Rao, Andrew Williams, Amit Patel

**Affiliations:** ^1^School of Medicine, University of Missouri-Kansas City, Kansas City, MO 64108, USA; ^2^Department of Medicine, Brooke Army Medical Center, San Antonio, TX 78234, USA; ^3^Department of Medicine—Division of Nephrology, Truman Medical Center, Kansas City, MO 64108, USA

## Abstract

Dialysis disequilibrium syndrome (DDS) is a morbid but rare complication of dialysis. Feared sequalae of this diagnosis are brain herniation and death. This report presents a patient who was diagnosed with DDS with subsequent tonsillar herniation shown on imaging with complete resolution of clinical signs and symptoms, as well as imaging findings of herniation after prompt initiation of intravenous mannitol. This is the first known case of reversal and survival of DDS-induced tonsillar herniation using mannitol.

## 1. Introduction

Dialysis disequilibrium syndrome (DDS) is a neurological complication of hemodialysis typically seen in individuals receiving therapy for the first time. First described in 1962, symptoms can include mild headache, vomiting, altered sensorium, seizures, arrhythmias, and even death [1]. Current research suggests that the reverse urea effect may be the primary mechanism due to which this syndrome occurs. Urea and other osmotically active solutes are critical in maintaining intravascular volume. During hemodialysis, these intravascular solutes are removed rapidly, creating an osmotic gradient that favors free water influx into the cerebral parenchyma [2]. This brisk expansion of tissue compresses pivotal brain structures and can result in cerebral herniation.

The diagnosis of DDS is one of the exclusions and may be difficult to identify by clinicians due to its vast array of presenting symptoms [3]. Luckily, most cases of DDS are mild and self-resolving, though a myriad of devastating consequences have been reported, including demyelination, posterior reversible encephalopathy syndrome, and most notably, cerebral herniation [4–6]. An extensive literature review yielded thirteen documented reports of patients suffering herniation secondary to DDS with poor outcomes. We present what we believe to be the first reported case of cerebellar tonsillar herniation secondary to DDS which was resolved using intravenous (IV) mannitol with no residual neurologic deficits.

## 2. Case Presentation

A 47-year-old African-American male with hypertension presented to the emergency department (ED) with complaints of persistent abdominal pain. Upon evaluation, the patient described his pain as vague and periumbilical. He also reported episodes of nonbloody emesis, headache, and possible hematuria since awaking that morning. His vital signs were significant for a blood pressure of 240/161 mmHg, heart rate of 100 beats per minute, respiratory rate of 18 breaths per minute, and oxygen saturation of 100% while he was breathing ambient air. He was afebrile. Physical exam revealed a malnourished-appearing male with an otherwise normal physical exam. Initial laboratory studies were significant for a creatinine of 15.39 mg/dL (reference range 0.90–1.3 mg/dL), blood-urea-nitrogen (BUN) of 155 mg/dL (reference range 8–20 mg/dL), an anion gap metabolic acidosis of 29 mmol/L (reference range 10–20 mmol/L), platelet count of 45 10^3^/cmm (reference range 150–400 10^3^/cmm), and hemoglobin of 10.1 g/dL (reference range 14.0–18.0 g/dL). Abdominal and pelvic computed tomography (CT) without contrast was performed and showed enteritis involving the jejunum.

The patient denied any history of renal or hematological disease. His home medications included labetalol 30 mg once per day and nifedipine 100 mg twice per day, both of which he admitted using sparingly. The patient was given intravenous (IV) labetalol in the ED with minimal success in lowering the blood pressure. Due to the presence of end-organ damage, hematological and metabolic aberrancies, thrombotic consumptive coagulopathy secondary to malignant hypertension was suspected. The patient was urgently admitted to the Intensive Care Unit (ICU) for further investigation and intervention.

In the ICU, a continuous IV infusion of nicardipine was initiated. Clinicians recommended urgent placement of a central venous catheter in order to initiate hemodialysis given electrolyte abnormalities. Initially, the patient refused all interventions due to holistic reasons. Fifteen hours following admission, the patient continued to deteriorate with worsening thrombocytopenia and encephalopathy; he ultimately agreed to catheter placement. Two units of platelets were transfused prior to the procedure. A right internal jugular vein triple lumen catheter was placed without complication, and the patient was initiated on hemodialysis with the dialysis prescription shown in Table 1.

A low-flux urea membrane was initially ordered by the attending nephrologist, but this option was not available at the facility in which this patient received treatment. Due to the urgency of treatment, hemodialysis was performed without this membrane.

**Figure 1:**
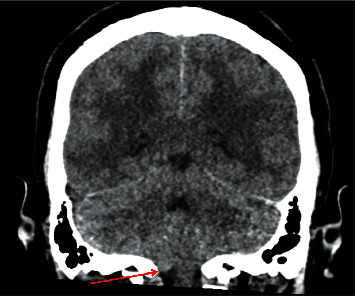
Computed Tomography (CT) showing cerebellar tonsil herniation (red arrow).

Approximately 20 minutes into the hemodialysis session, the patient became unresponsive. A right eye gaze deviation and right-sided fasciculations of the upper and lower extremities were observed. Hemodialysis was discontinued, and an urgent head CT without contrast was performed. Imaging revealed pontine edema with mass effect and cerebellar tonsillar herniation (Figure 1). The on-call nephrologist initiated intravenous mannitol, and neurosurgery was consulted urgently. The results of complete basic metabolic panels before and after the initiation of hemodialysis are shown in Table 2.

Within 30 minutes of initiation of the mannitol infusion, the patient regained consciousness and remained hemodynamically stable. Follow-up neurological exam showed no focal deficits and complete resolution of the right-sided fasciculations and gaze palsy. Mannitol was continued for 24 hours. The following morning, head magnetic resonate imaging (MRI) was obtained and showed resolution of the midline shift and tonsillar herniation shown on the head CT scan eight hours prior. A diagnosis of Dialysis Disequilibrium Syndrome was made.

Three days following this diagnosis, a low-flux urea filter was obtained from an outside facility and the patient began continuous renal replacement therapy (CRRT). Serial head CT occurring every 48 hours and scheduled hourly neurologic examinations confirmed the patient's tolerance of CRRT with no further progression or recurrence of symptoms related to DDS. The patient was eventually transferred out of the ICU and underwent placement of a long-term hemodialysis fistula. He had complete resolution of symptoms at the 2-year follow-up, but remained on intermittent hemodialysis.

## 3. Discussion

While the mechanism of DDS remains unclear, several risk factors for the syndrome have been proposed, including first dialysis, extremes of age, electrolyte imbalances, metabolic acidosis, hypertensive emergency, pre-existing neurological conditions, cerebral edema, meningitis, and the presence of intracranial tumors [7]. Additionally, the hemodialysis prescription prescribed by the provider may influence a fluid shift into the cerebral parenchyma. Current guidelines recommend limiting the removal of BUN to 40% over the course of two hours. This can be achieved by utilizing low-flux dialysis membranes, setting low flow rates (Qd of 300 and Qb 250, respectively), modulating the dialysate sodium and glucose concentrations, and shortening the duration of therapy. Additional methods, including prophylactic mannitol administration, have been suggested [2, 7, 8].

As described in the case above, our patient had several predisposing risk factors for DDS upon presentation to the ED. He was in hypertensive emergency and had a metabolic acidosis and significantly elevated BUN. This was also his first-time dialyzing. When dialysis was initiated, the duration was shortened to 120 minutes, dialysate sodium was set at 140 mmol/L, and there was an attempt to secure a low-flux dialysis membrane. Unfortunately, even with these adjustments to therapy, the patient cleared 31 mg/dL of BUN or 20% of his total serum BUN in 20 minutes, as seen by comparing the patient's initial metabolic panel to the one drawn thirty minutes after initiation of HD (Table 2). Other interventions, such as the addition of dialysate glucose, increasing dialysate sodium further, utilizing lower Qb and Qd flow rates, and administering prophylactic mannitol, may have been beneficial at reducing the likelihood of DDS occurrence in this patient.

Historically, even with swift intervention, cerebral herniation due to DDS carries a grim prognosis. A case series by Osgood et al. in 2015 highlighted that four patients who all received hemodialysis were diagnosed with DDS and had cerebral herniation confirmed on imaging. Despite intervention with mannitol and hypertonic saline, all patients succumbed to their neurologic injury and were pronounced brain dead [6]. Similarly, a case series published by Kumar et al. in 2014 highlighted a patient suffering from DDS with resultant tonsillar herniation. Despite timely introduction of an infusion of hypertonic saline, the patient died [9]. With the exception of the case reported above, an extensive literature review failed to reveal any other documented cases of survival (with or without return to baseline) among patients with DDS and imaging-confirmed tonsillar herniation.

Guideline-based management of cerebral herniation secondary to DDS currently recommends the cessation of HD, hyperventilation, and elevating the head of the bed. Although numerous studies have shown mannitol's efficacy at treating a variety of neurological conditions, the use of mannitol and other osmotically active infusions for the management of DDS remains controversial [10, 11]. This report is the first known case in which a mannitol infusion successfully reversed DDS-induced tonsillar herniation with a complete return to neurologic baseline.

## 4. Conclusions

The case reported herein is the first documented account of reversal of the clinical and imaging findings of tonsillar herniation secondary to DDS using intravenous mannitol.

## Figures and Tables

**Table 1:** Dialysis prescription.

F180 membrane	
Surface area	1.7 m^2^
KUf	58 mL/hr/mmHg

Dialysate solution	
Sodium	140 mmol/L
Potassium	4 mmol/L
Calcium	2.5 mmol/L
Bicarbonate	35 mmol/L

Q_D_	600 mL/min

Q_B_	300 mL/min

**Table 2:** Results of basic metabolic panels.

	Predialysis	Postdialysis
Sodium	135 mmol/L	137 mmol/L
Potassium	3.2 mmol/L	3.2 mmol/L
Chloride	89 mmol/L	91 mmol/L
Bicarbonate	22 mmol/L	26 mmol/L
Blood-Urea-Nitrogen	152 mg/dL	121 mg/dL
Creatinine	16.25 mg/dL	11.75 mg/dL
Glucose	175 mg/dL	130 mg/dL
